# Short term synaptic depression with stochastic vesicle dynamics imposes a high-pass filter on presynaptic information

**DOI:** 10.1186/1471-2202-13-S1-O17

**Published:** 2012-07-16

**Authors:** Robert Rosenbaum, Jonathan Rubin, Brent Doiron

**Affiliations:** 1 Mathematics, University of Pittsburgh, Pittsburgh, PA 15260, USA; 2 Center for the Neural Basis of Cognition, Pittsburgh, PA, 15213, USA

## 

The filtering properties of synapses are modulated by a form of short term depression arising from the depletion of neurotransmitter vesicles. The uptake and release of these vesicles is stochastic in nature, but a widely used model of synaptic depression does not take this stochasticity into account. While this model of synaptic depression accurately captures the trial-averaged synaptic response to a presynaptic spike train [1], it fails to capture variability introduced by stochastic vesicle dynamics [2]. Our goal is to understand the impact of stochastic vesicle dynamics on filtering and information transfer in depressing synapses.

**Figure 1:**
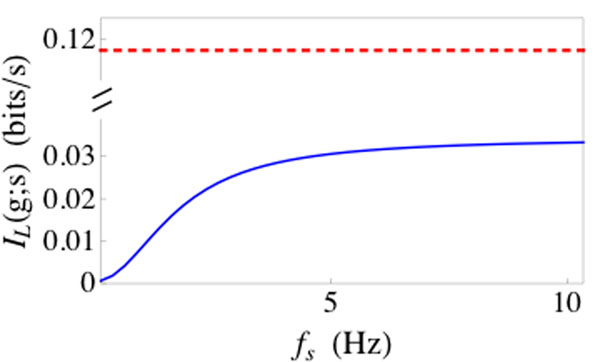
The linear information rate, *I_L_*(*g;s*), which represents the information per unit time available to an optimal linear decoder that estimates a rate-coded presynaptic signal, *s*(*t*), by observing a postsynaptic conductance, *g*(*t*). The linear information rate is plotted as a function of the peak frequency, *f_s_*, of the signal. When stochastic vesicle dynamics are ignored (dashed red line), *I_L_*(*g;s*) is independent of *f_s_*[3,4]. When stochastic vesicle dynamics are accounted for (solid blue line), information transfer is reduced and high-frequency signals are transferred more reliably than low-frequency signals.

We derive compact, closed-form expressions for the synaptic filter induced by short term synaptic depression when stochastic vesicle dynamics are taken into account and when they are not. We find that stochasticity in vesicle uptake and release fundamentally alters the way in which a synapse filters presynaptic information. Predictably, the variability introduced by this stochasticity reduces the rate at which information is transmitted through a synapse. Additionally, this variability introduces frequency-dependence to the transfer of information through a synapse: a model that ignores synaptic variability transmits slowly varying signals with the same fidelity as faster varying signals [3,4], but a model that takes this variability into account transmits faster varying signals with higher fidelity than slower signals (Figure 1). Differences between the models persist even when the presynaptic cell makes many contacts onto the postsynaptic cell. We extend our analysis to the population level and conclude that a slowly varying signal must be encoded by a large presynaptic population if it is to be reliably transmitted through depressing synapses, but faster varying signals can be reliably encoded by smaller populations. Our results provide useful analytical tools for understanding the filtering properties of depressing synapses and have important consequences for neural coding in the presence of short term synaptic depression.
